# PLAG1 interacts with GPX4 to conquer vulnerability to sorafenib induced ferroptosis through a PVT1/miR-195-5p axis-dependent manner in hepatocellular carcinoma

**DOI:** 10.1186/s13046-024-03061-4

**Published:** 2024-05-14

**Authors:** Jiarui Li, Yilan Li, Denghui Wang, Rui Liao, Zhongjun Wu

**Affiliations:** Department of Hepatobiliary Surgery, the First Affiliated Hospital of Chongqing Medical University, Chongqing, 400010 China

**Keywords:** Hepatocellular carcinoma, Sorafenib, PLAG1, Ferroptosis, GPX4

## Abstract

**Background:**

Sorafenib is a standard first-line treatment for advanced hepatocellular carcinoma (HCC), yet its effectiveness is often constrained. Emerging studies reveal that sorafenib triggers ferroptosis, an iron-dependent regulated cell death (RCD) mechanism characterized by lipid peroxidation. Our findings isolate the principal target responsible for ferroptosis in HCC cells and outline an approach to potentially augment sorafenib's therapeutic impact on HCC.

**Methods:**

We investigated the gene expression alterations following sgRNA-mediated knockdown induced by erastin and sorafenib in HCC cells using CRISPR screening-based bioinformatics analysis. Gene set enrichment analysis (GSEA) and the "GDCRNATools" package facilitated the correlation studies. We employed tissue microarrays and cDNA microarrays for validation. Ubiquitination assay, Chromatin immunoprecipitation (ChIP) assay, RNA immunoprecipitation (RIP) assay, and dual-luciferase reporter assay were utilized to delineate the specific mechanisms underlying ferroptosis in HCC cells.

**Results:**

Our study has revealed that pleiomorphic adenoma gene 1 (PLAG1), a gene implicated in pleomorphic adenoma, confers resistance to ferroptosis in HCC cells treated with sorafenib. Sorafenib leads to the opposite trend of protein and mRNA levels of PLAG1, which is not caused by affecting the stability or ubiquitination of PLAG1 protein, but by the regulation of PLAG1 at the transcriptional level by its upstream competitive endogenous long non-coding RNA (lncRNA) plasmacytoma variant translocation 1 (PVT1). Data from 139 HCC patients showed a significant positive correlation between PLAG1 and GPX4 levels in tumor samples, and PLAG1 is instrumental in redox homeostasis by driving the expression of glutathione peroxidase 4 (GPX4), the enzyme that reduces lipid peroxides (LPOs), which further leads to ferroptosis inhibition.

**Conclusions:**

Ferroptosis is a promising target for cancer therapy, especially for patients resistant to standard chemotherapy or immunotherapy. Our findings indicate that PLAG1 holds therapeutic promise and may enhance the efficacy of sorafenib in treating HCC.

**Supplementary Information:**

The online version contains supplementary material available at 10.1186/s13046-024-03061-4.

## Introduction

HCC is the second most common cause of cancer-related deaths globally, largely due to late diagnosis and its links to lifestyle factors such as alcohol consumption, and conditions like metabolic syndrome and viral infections [1]. One major issue with HCC treatment is the low efficacy rate of sorafenib, an FDA-approved drug [2], which shows favorable responses in only 30% of HCC patients. Furthermore, resistance to sorafenib often develops within a year [3]. Understanding the molecular mechanisms behind sorafenib resistance and identifying new targets are critical for advancing cancer therapeutics and increasing patient survival rates.

Sorafenib, an oral multi-kinase inhibitor, has been shown benefits against various tumors, notably in liver cancer. Its efficacy results from its ability to inhibit multiple kinases, leading to halted tumor cell growth, restricted angiogenesis, and induced apoptosis [4, 5]. However, acquired or intrinsic resistance to apoptosis can limit the effectiveness of sorafenib-induced cell death. Interestingly, sorafenib also triggers ferroptosis, a newly recognized form of RCD, in hepatoma cells [6, 7]. This action isn't based on its multi-kinase inhibitory function and enhances the drug's anti-cancer effect [8]. As opposed to regular apoptosis and necrosis, ferroptosis is caused by cellular contraction and increased mitochondrial membrane density, consequently to the intracellular buildup of iron-dependent lipoperoxidation. Therefore, encouraging ferroptosis via sorafenib represents a potential new direction in liver cancer treatment enhancement.

PLAG1, a gene located on chromosome 8q12, consists of 5 exons and has been linked to cancer-related activities such as proliferation, migration, and invasion [9, 10]. The gene bears crucial similarities with PLAGL2, both structurally and functionally. In liver cancer, PLAGL2 enhances the activation of hypoxia-inducible factor 1 (HIF1). This factor promotes the transcription of the SLC7A11 subunit of the cystine/glutamate antiporter system Xc(-), effectively preventing the onset of ferroptosis [11, 12]. PLAG1 contains seven typical C2H2 zinc finger structures, highlighting its potential transcription factor role, particularly through its COOH-terminal domain's trans-activation ability [13]. However, the role of PLAG1 in the regulation of sorafenib-induced ferroptosis in liver cancer remains a subject for further investigation.

The enzyme GPX4 is crucial for the clearance of LPOs [14]. This process relies on the system Xc(-), which transports cystine into the cell, an essential component for glutathione (GSH) synthesis during cellular scavenging activities [15]. GPX4 uses glutathione to reduce LPOs into phospholipid molecules, making its role vital in boosting the cell's antioxidant capacity, which helps protect liver cancer cells from ferroptosis. Consequently, focusing on manipulating GPX4 could offer a potential strategy in enhancing tumor therapy, particularly in overcoming sorafenib resistance.

Our study indicates the impact of PLAG1 on ferroptosis inhibition in the case of sorafenib treatment, is determined by the regulation of the PVT1/miR-195-5p axis. Additionally, a positive correlation between PLAG1 and GPX4 expression in HCC patients and a crosstalk between PLAG1 and GPX4 in ferroptosis inhibition are due to PLAG1's ability to amplify GPX4 gene transcription by binding to its promoter region. In conclusion, our research clarifies the role of PLAG1 in ferroptosis and its potential as a responsive therapeutic target for HCC patients resistant to sorafenib treatment.

## Materials and methods

### Cell lines and culture

The liver cancer cell lines (HCCLM9, HCCLM3, SK-hep1, MHCC97H, and MHCC97L) and the normal hepatocyte MIHA were obtained from Shanghai Institutes of Biological Sciences (Shanghai, China). They were cultured in 90% DMEM (ZQXZ-bio, Shanghai, China) supplemented with 10% fetal bovine serum (SORFA, Beijing, China) at a temperature of 37 °C with 5% CO_2_.

### Lentivirus production

Hanbio Biotechnology (Shanghai, China) provided lentivirus vectors that include shRNA to silence PLAG1 (targeting GGAGCACCUUAAAUCUCAUTT), lentiviruses that overexpress PLAG1 (LV-PLAG1), shRNA to silence PVT1 (targeting GGCCTCGTGTCTATTAAAT), and a negative control lentivirus. Genechem (Shanghai, China) sold lentiviruses called LV-PVT1, which overexpressed PVT1, as well as control lentiviruses. The MHCC97H (MOI = 5) or MHCC97L (MOI = 30) cell lines were seeded in six-well plates with 150,000 cells per well, followed by lentiviral infection on the following day. To establish stably infected cell lines, 2 μg/ ml puromycin was applied after infection 72 h for at least two weeks.

### Cell transfection

Tsingke Biotechnology (Beijing, China) provided the expression vector pcDNA3.1( +) (PVT1) and the empty plasmid pcDNA3.1( +) (Vector). The PVT1 siRNA (which targets GGCCTCGTGTCTATTAAAT) was acquired from GenePharma (Suzhou, China). RiboBio (Guangzhou, China) sold the hsa-miR-195-5p mimic (miR10000461–1–5) and hsa-miR-195-5p inhibitor (miR20000461–1–5) that were acquired. The cell transfection was carried out in accordance with the guidelines provided by the manufacturers.

#### cDNA Microarray and quantitative real‑time polymerase chain reaction (qRT‑PCR)

We purchased the cDNA microarray (MecDNA-HLivH087Su02 and MicDNA-HLivH087Su02) from Shanghai Outdo Biotech Co. The FastPure Cell/Tissue Total RNA Isolation Kit V2 (Vazyme, Nanjing, China) was utilized for the purification of total RNA. The PrimeScript™ RT reagent Kit (Takara, Japan) was used to convert 1 μg total RNA into cDNA through reverse transcription. For the examination of miRNA expression, we utilized the Hairpin-it™ miRNA Quantitation Kit (GenePharma, Suzhou, China) along with reverse transcription using specific stem-loop primers for miR-20a-5p, miR-195-5p, miR-17-5p, miR-106b-5p, and miR-93-5p. qRT-PCR was conducted using SYBR Green (Selleck, USA). The parameters for the two-stage amplification process were as follows: initial denaturation at a temperature of 95 °C for a duration of 2 min, followed by 40 cycles of denaturation at 95 °C for 15 s and annealing at 60 °C for 30 s. GAPDH and U6 expression served as the internal reference for mRNA/lncRNA and miRNA, respectively. Supplementary Table 1 displays the specific primers for RNAs.

### Immunoblotting

The cells were gathered and broken down in M-PER (R) Mammalian Protein Extraction Reagent (Thermo Fisher Scientific, USA) while being kept on ice for a duration of 30 min. The cell lysates underwent resolution through SDS-PAGE and were subsequently transferred to PVDF membranes (Cytiva, USA). The PVDF filters were treated with QuickBlock™ Western blocking solution (Beyotime, China) for 1 h while gently shaking. Subsequently, the filters were incubated with the specified antibodies at 4 °C overnight. Goat secondary antibodies conjugated with HRP (1:1000, Beyotime, China) were employed. After being washed with TBST, the membranes underwent a thorough cleansing, followed by incubation of the secondary antibody at room temperature for 2 h. To examine the protein bands, we employed a chemiluminescent western blot detection method.

### Tissue microarray and immunohistochemistry staining

AiFang Biological (Changsha, China) generated TMA using 139 paraffin-embedded samples. Immunohistochemistry (IHC) Kit (Absin, Shanghai, China) was employed to identify the correlation between PLAG1 and GPX4 expression in hepatocellular carcinoma tissues. For IHC staining, the antibodies employed were anti-PLAG1 (1:100, Novus Biologicals, USA) and anti-GPX4 (1:50, Selleck, USA). Two independent pathologists blinded to the clinical data evaluated the PLAG1 and GPX4 IHC staining using a histological score (H-score) approach. IHC analysis was conducted following the previously mentioned protocol [16]. Briefly, the staining intensity was classified into the following categories: 0 (-), 1 ( +), 2 (+ +), and 3 (+ + +). The mean proportion of cells with positive staining was assessed using the following scoring system: 0 (< 5%); 1 (5–25%); 2 (26–50%); 3 (51–75%) and 4 (76–100%). The overall scores were calculated by multiplying the scores for staining intensity with the scores for staining proportion, which ranged from 0 to 12. To divide them into low and high expression subgroups, the median H-scores of PLAG1 and GPX4 were utilized as thresholds.

### Antibodies and molecular compounds

The antibodies utilized in this investigation for western blot and immunohistochemistry analysis were as follows: PLAG1 (H00005324-M02, Novus Biologicals, 1:500 for WB, 1:50 for IHC), GPX4 (A5569, Selleck, 1:1000 for WB), AGO2 (#2897, Cell Signaling Technology, 1:1000 for WB), GAPDH (60,004–1-lg, Proteintech, 1:50,000 for WB), 4-HNE (MHN-020P, JalCA, 1:4 for IHC), Ubiquitin(AF1705, Beyotime, 1:1000 for WB). Beyotime and Selleck Chemicals were the sources of the secondary antibody conjugates labeled with Horseradish peroxidase (HRP) and Sorafenib (#S7397), respectively. Cycloheximide (CHX, HY-12320) and MG132 (HY-13259) were purchased from Medchemexpress.

### Assessment of cellular viability

The CCK8 (Absin, Shanghai, China) was utilized to assess cellular viability. In short, cells were seeded onto 96-well plates at a density of 5,000 cells per well. Once the cells reached 60% confluence, they were treated with either sorafenib (10 μM) or dimethyl sulfoxide (DMSO) for a duration of 24 h. Following this, 10 μL of CCK-8 solution was introduced into each well and incubated for 2 h. Subsequently, the absorbance at 450 nm was determined using a microplate reader.

### Investigation of ferroptosis

(1) To determine the Malondialdehyde (MDA) levels, 5 × 10^6^ cell lysates were analyzed using a Cell Malondialdehyde Assay Kit (A003-4–1, Jiancheng Bio) following the provided protocol. MDA reacts with thiobarbituric acid (TBA) to produce a crimson MDA-TBA compound, exhibiting a peak absorbance at 532 nm.

(2) The GSH test involved seeding corresponding cells in 6 cm plates at a density of 3 × 10^6^ per plate. These cells were then treated with either sorafenib (10 μM) or DMSO for a duration of 48 h. Afterward, the cells were washed and collected using PBS. The lysates were analyzed with a commercially available Reduced GSH Assay Kit (A006-2–1, Jiancheng Bio). Yellow product with maximum light absorption at wavelength 412 nm, and its absorbance is proportional to GSH content.

(3) To identify lipid peroxidation products, we utilized LPOs analysis with Liperfluo (Dojindo, Japan). In short, cells were placed on a glass bottom dish with a diameter of 35 mm (5 × 10^4^ cells/dish). After 24 h, the culture medium was exchanged with sorafenib (10 μM) or DMSO and incubated for another 24 h. Following that, 10 µM Liperfluo probes were applied to label LPOs at a working concentration, and incubated for 30 min. Finally, the cells were observed and photographed using confocal microscopy.

(4) To measure intracellular Fe^2+^, a total of 1 × 10^6^ cells was rinsed three times with cold PBS (Procell, Wuhan), gathered, suspended, and then exposed to 1 μM FerroOrange probes (Dojindo, Japan) for 30 min at 37 °C. Subsequently, flow cytometry analysis was conducted using Ex 561 nm/Em 570–620 nm.

(5) The Lipid Reactive Oxygen Species (ROS) assay involved incubating 1 × 10^6^ cells with 5 μM BODIPY-C11 (Glpbio, USA) at 37 °C for 30 min. The cells were collected and placed in DMEM without serum, then analyzed using flow cytometry or confocal microscopy (with an excitation wavelength of 488 nm and an emission wavelength range of 510–555 nm).

### Chromatin immunoprecipitation (ChIP) and truncation assay

An Enzymatic ChIP Kit (Cell Signaling Technology, USA) was utilized for conducting a ChIP assay. JASPAR (https //jaspar.genereg.net) provided the top seven potential sequences between PLAG1 and the GPX4 promoter (-2000 bp ~  + 99 bp). We truncated the GPX4 promoter region and designed primers for each segment that contains the binding site, based on the anticipated distribution of sites in the GPX4 promoter region. Formaldehyde was added to cross-link MHCC97H cells, and glycine was added to terminate the process. Sonication was used to produce fragments ranging from 200 to 900 bp in length from lysates. Specific DNA–protein complexes were immunoprecipitated using an incubating antibody (anti-PLAG1 and lgG) (Novus Biologicals, USA). In addition, MHCC97H cells were plated onto 10 cm dishes and co-transfected with either the PLAG1 overexpression vector or the normal control (NC) groups. The binding of the GPX4 core promoter region (-250 bp ~  + 120 bp) to RNA polymerase II (Pol II) was verified using the same method. The quantification and analysis of purified DNA was performed using qRT-PCR. Supplementary Table 2 displayed the details of the primers.

#### Construction of competing endogenous RNA (ceRNA) network and the luciferase reporter assay

From TCGA-LIHC, we detected the distinct expression of miRNAs, mRNAs, and lncRNAs. To build the ceRNA network of lncRNA-miRNA-mRNA, we utilized the GDCRNATools R package [17]. Cytoscape v3.6.0 [18] was utilized to generate the ceRNA network. We utilized three different target prediction algorithms, namely miRcode [19], starBase [20], and spongeScan [21], to explore the potential miRNAs associated with crosstalk with PVT1. Upon identifying miR-195-5p as the focus of our study, we proceeded to discover and forecast the probable binding locations of miR-195-5p in the 3′-UTR regions of PVT1 and PLAG1. The luciferase reporter vectors PVT1-wild type (WT) and PVT1-mutant (MUT) were created using PmirGLO from Promega, an American company. The introduction of mutations in the potential binding sites of miR-195-5p was carried out using the QuickMutation™ Site-Directed Mutagenesis Kit from Beyotime, a company based in China.24-well plates were used to seed HEK293T cells, which were then co-transfected with PVT1-WT or MUT vectors along with miR-195-5p mimic or miR-NC.Using the identical approach, the confirmation of the connection between miR-195-5p and the 3′-UTR portions of PLAG1 was established.The luciferase activity was evaluated using a luciferase reporter assay kit (GenePharma, China) following the instructions provided by the manufacturers.

### RNA immunoprecipitation (RIP)

RIP is a technique used to isolate RNA molecules. RNA immunoprecipitation kit (Geneseed, Guangzhou, China) was used to detect the interaction of PVT1 and miR-195-5p, as well as PLAG1 and miR-195-5p, following the instructions provided by the manufacturer. In brief, around 2 × 10^7^ MHCC97H cells were transfected with miR-195-5p mimics or miR-NC using the riboFECT CP Transfection Kit (RiboBio, Guangzhou, China). After being treated, the cells were suspended and broken down in RIP lysis solution. The cell lysates were mixed with RIP immunoprecipitation buffer that had magnetic beads attached to anti-Ago2 antibody (Cell Signaling Technology, USA) and control IgG antibody (Cell Signaling Technology, USA). This mixture was then incubated overnight at a temperature of 4 °C. Following incubation with Proteinase K, the RNA that was immunoprecipitated was eluted, isolated, and measured using qRT-PCR and western blotting.

### *Fluorescence *in Situ* Hybridization (FISH)*

FISH is a technique that can be used to detect and locate specific DNA/RNA sequences in cells. The location of PVT1 in MHCC97H and MHCC97L cells was observed using the FISH assay. Probes labeled with Cy5 for PVT1 were synthesized by Servicebio (Wuhan, China). HCC cells were grown on coverslips and treated with a FISH probe in hybridization buffer (Servicebio, Wuhan, China) for 16 h at 37 °C. The cell nuclei were then stained with DAPI (4′6-diamidino-2-phenylindole). The Olympus BX51 fluorescence microscope (Tokyo, Japan) was used to capture these images. Supplementary Table 3 contained the displayed probe sequences.

### Transmission electron microscopy

The protocol for the transmission electron microscope was executed according to the previously stated description [22]. In short, MHCC97H cells were cultured on a 60 mm dish with a density of 6 × 10^5^ cells per dish and incubated for 24 h prior to treatment. MHCC97H cells were fixed with 2.5% glutaraldehyde at 4 °C for 24 h and treated with sorafenib (10 μM) or DMSO for 24 h. Subsequently, the cells were exposed to 1% osmium tetraoxide for 2 h after drug treatment. Dehydration of the cells lasted for 15 min before embedding them in resin. Using a JEM-1400 electron microscope (JEOL Ltd., Japan), representative images were acquired after slicing and double-staining the samples with uranyl acetate and lead citrate.

### Bioinformatics

Data on gene expression and patient information for hepatocellular carcinoma were acquired from the TCGA website (https//cancergenome.nih.gov/). Using the DESeq2 package of R software (version 4.2.1) [23], we determined the variation in counts within the dataset GSE182185 and GSE109211 obtained from the GEO website (https//www.ncbi.nlm.nih.gov/). The difference analysis results were then visualized using the ggplot2, where the horizontal axis represented the position after log (FC) was sorted and the vertical axis represented log (FC). The Hi-C and ChIP-seq data were downloaded from datasets GSE184796 and GSE151287. Initially, the unprocessed sequencing data undergoes quality control procedures, which involve evaluating the quality of the reads and eliminating reads with low quality. Afterwards, the sequencing data is aligned to the reference genome using bwa (v0.7.17) in order to identify their genomic locations [24]. From the mapped reads, a contact matrix or a bipartite graph is constructed, where the matrix elements or graph edges represent the frequency of interactions between genomic loci. Normalization techniques are then applied to correct for biases arising from sequencing depth and chromosome length. Subsequently, topologically associated domains (TADs) can be identified using juicer software (http://aidenlab.org/juicer/), partitioning the genome into regions with similar intra-chromosomal interaction patterns. The juicer (v3.0.0) can be employed to visualize the contact matrix, TADs, and other structural features [25].

### Animal studies

GemPharmatech (Jiangsu, China) supplied BALB/c nude mice that were 4 weeks old and female. (1) To establish the subcutaneous mouse model, around 5 × 10^6^ MHCC97H/sh-NC cells or MHCC97H/sh-PLAG1 cells are surgically inserted under the skin on the right side. Upon reaching a tumor volume of 50 mm^3^, mice harboring MHCC97H/sh-NC cells or MHCC97H/sh-PLAG1 cells were randomly allocated into two groups. One group received vehicle treatment (0.9% NaCl i.g., once daily), while the other group received sorafenib treatment (10 mg/kg i.g., once daily) for a duration of two weeks. Measurements of tumors were taken every second day. The formula for calculating tumor volume is V = (L × W^2^) / 2, where V denotes the volume of the tumor, L represents the tumor's length, and W represents its width. Following a 14-day period of treatment, the mice were euthanized and the tumors were excised.

(2) To establish the orthotopic mouse model, initially, around 5 × 10^6^ MHCC97H/sh-NC cells or MHCC97H/sh-PLAG1 cells were subcutaneously injected into the right flank. Afterwards, we proceeded to dissect the tumors and divide them into 1 mm^3^ cubes under sterile conditions on day 14. After segmenting the tumors, they were implanted into the liver parenchyma of female BALB/c nude mice that were 4 weeks old, specifically in the right lobe. On the seventh day, mice carrying MHCC97H/sh-NC cells or MHCC97H/sh-PLAG1 cells were randomly separated into two groups. One group received treatment with a solution of 0.9% NaCl (i.g., once daily), while the other group was administered sorafenib at a dose of 10 mg/kg (i.g., once daily). After 21 days of implantation, the mice were sacrificed and the tumors were dissected. For subsequent IHC experiments, fixation with 4% paraformaldehyde was employed. The tumor volumes were then determined using the identical formula as previously.

### Statistical analysis

At least three independent experiments were conducted and the data were presented as the mean plus or minus the standard deviation (± SD). The data from the experiments were examined using either the two-tailed Student's t-test or ANOVA in GraphPad Prism 8. We utilized the Pearson χ^2^ test for comparing qualitative variables. *P* value < 0.05 were considered statistically significant.

## Results

### The treatment with sorafenib in HCC leads to a decrease in the oncoprotein PLAG1

**Fig. 1:**
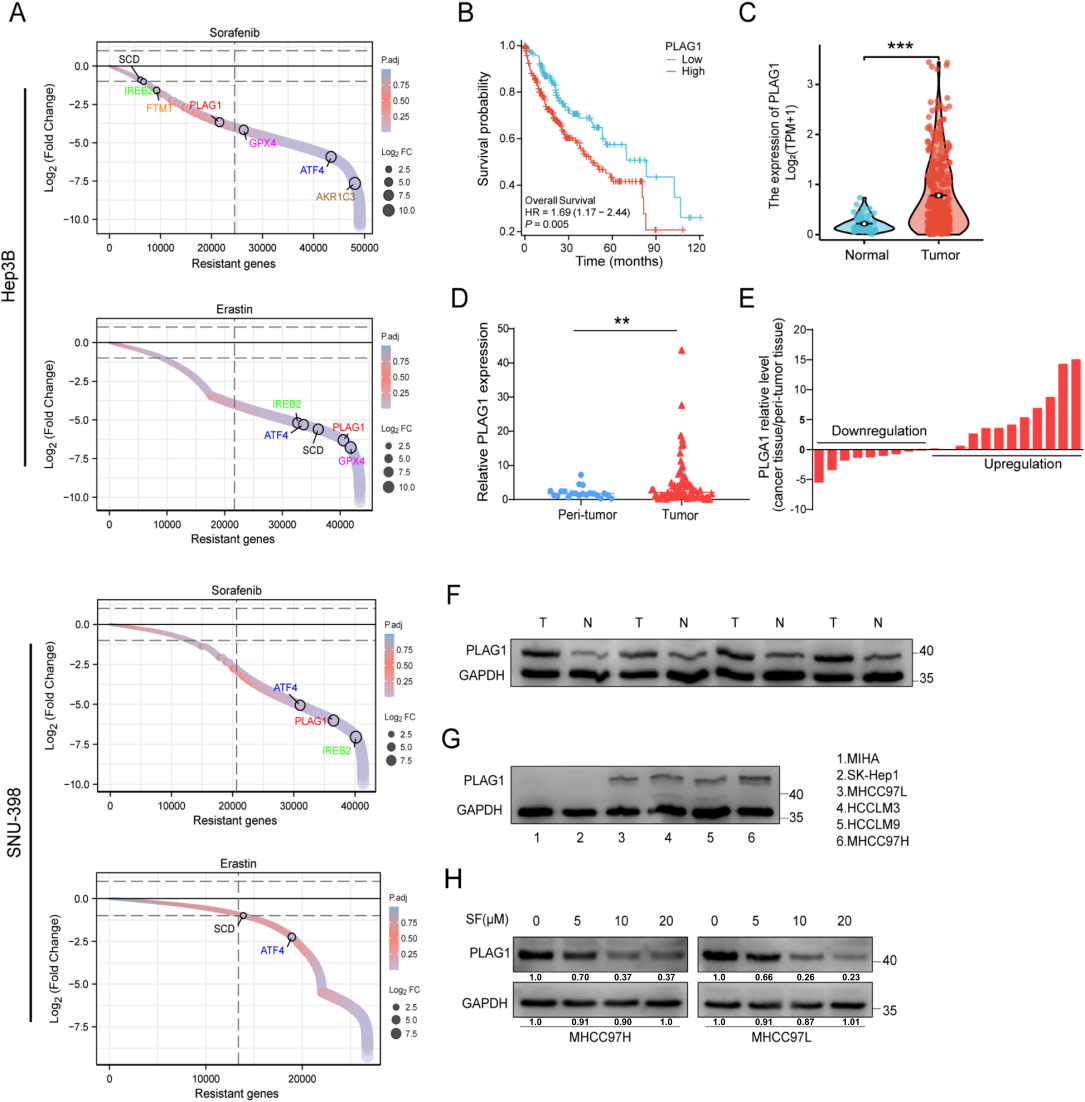
The administration of sorafenib in HCC patients results in a decrease in the oncoprotein PLAG1. (**A**) Sorting charts depicting alterations in reanalyzing of CRISPR screen results, with genes related to ferroptosis exhibiting significant depletion, as indicated by colored dots. Notably, the treatment with erastin and sorafenib leads to a considerable reduction in PLAG1 levels in hepatoma cells. (**B**) Kaplan–Meier graphs illustrating the negative correlation between PLAG1 mRNA levels and the survival outcomes of 374 HCC patients obtained from the TCGA database. A comparative analysis was conducted to assess the expression of PLAG1 in HCC and healthy tissues. Data from the TCGA database (**C**) and cDNA microarray (**D**) were utilized for this purpose. (**E**) The qRT-PCR technique was employed to measure the levels of PLAG1 mRNA expression in 21-paired tumor and peritumoral tissues obtained from HCC patients. The obtained data were presented as − ΔΔCt values. To assess the expression levels of PLAG1 protein, a western blot analysis was conducted on four hepatocellular carcinoma (HCC) tissues and their corresponding peritumoral tissues (**F**), as well as on MIHA, SK-Hep1, MHCC97L, HCCLM3, HCCLM9, and MHCC97H cell lines (**G**). The results showed that the expression of PLAG1 was lower in MHCC97L cells and higher in MHCC97H cells. Therefore, MHCC97L cells and MHCC97H cells were used for subsequent experiments. Subsequently, the protein levels of PLAG1 in MHCC97H and MHCC97L cell lines were determined using western blot analysis, following treatment with DMSO or sorafenib (SF) for 24 h in a dose-dependent manner (**H**). Statistical analysis revealed significant differences with **P < 0.01 and ***P < 0.001

Our bioinformatics analysis, employing CRISPR-based screening, revealed a marked decrease in sgRNA targeting the oncogenic protein PLAG1 in HCC cells following treatment with ferroptosis inducers erastin and sorafenib. This suggests that PLAG1 potentially contributes to ferroptosis resistance (Fig. 1A). Kaplan–Meier analysis of overall survival rates concerning 374 patients from the TCGA-LIHC cohort highlighted an inverse relationship between PLAG1 levels and survival rates, indicating its contribution to HCC progression (Fig. 1B). A more localized study using qRT-PCR demonstrated an elevation in PLAG1 mRNA levels in 87 tumor tissues compared to normal tissues, consistent with data from the TCGA-LIHC RNA sequencing and cDNA-microarray analysis (Fig. 1C-E). This result was further confirmed by paired samples from HCC patients (Fig. 1F). Western blotting and qRT-PCR showed that PLAG1 was upregulated in MHCC97L, HCCLM3, HCCLM9, and MHCC97H cells, with the lowest expression in MHCC97L cells and the highest expression in MHCC97H cells compared with normal hepatocyte MIHC (Fig. 1G and Supplementary Fig. 1). Therefore, we selected MHCC97L and MHCC97H for follow-up studies. Fascinatingly, sorafenib dosage demonstrated a proportional suppression of the PLAG1 level in HCC cell lines MHCC97H and MHCC97L suggesting its potential to combat PLAG1 expressions in HCC progression (Fig. 1H). These findings emphasize the significant role of PLAG1 in the progression of HCC, and its potential as a therapeutic target.

### PLAG1 plays a role in inhibiting ferroptosis in HCC cells treated with sorafenib

**Fig. 2:**
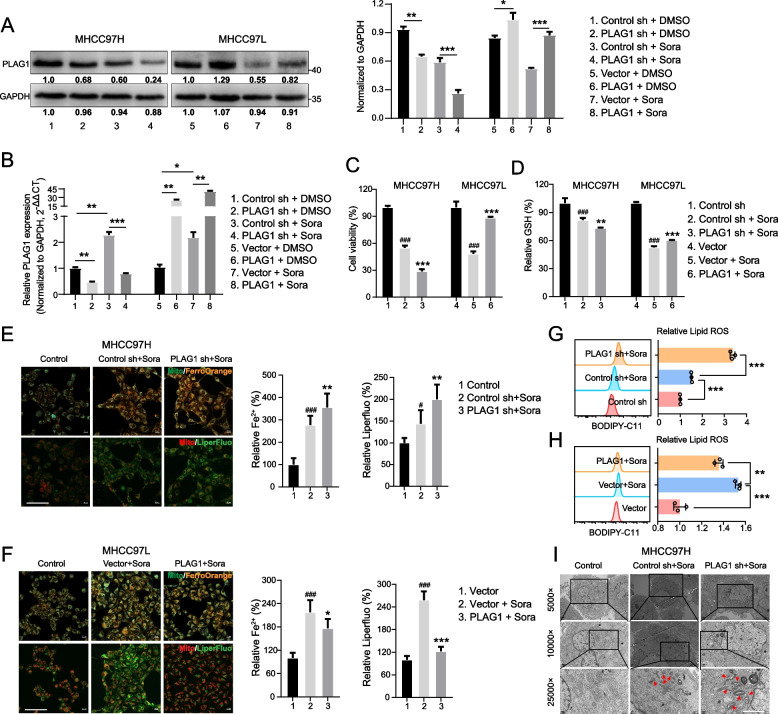
PLAG1 inhibits sorafenib-induced ferroptosis. (**A**) Western blot and qRT-PCR were used to detect the relative expression of PLAG1 in MHCC97H and MHCC97L cells transfected with the specified constructs. Transfected cells were exposed to sorafenib (10 µM) for 24 h. Cell viability was assessed using a CCK-8 kit (**C**). The concentration of GSH in the cells was measured using a glutathione assay kit (**D**). Intracellular Fe^2+^ levels were determined using the fluorescent probe FerroOrange, while lipid peroxide levels were measured using the fluorescent probe Liperfluo (**E**, **F**). Scale bar: 100 μm. (**G**, **H**) Lipid ROS accumulation was analyzed by flow cytometry with BODIPY-C11 staining. (**I**) The transmission electron microscopy was used to observe the structure of mitochondria and showed that knockdown of PLAG1 could promote the increase of mitochondrial electron density and mitochondrial shrinkage. Scale bar: 2μm. The data displayed indicate the mean ± SD obtained from three independent trials. ^#^*P* < 0.05; ^###^*P* < 0.001 compared with the control group. *P < 0.05; **P < 0.01; ***P < 0.001 compared with the sorafenib-induced control group

Evidence increasingly suggests ferroptosis's role in sorafenib-induced cell death in HCC [6, 26, 27]. This study aimed to ascertain PLAG1's role in ferroptosis inhibition. To this end, HCC cells with PLAG1 knockdown and overexpression were respectively generated in cell MHCC97H and MHCC97L, then treated with sorafenib. Western blot and qRT-PCR analysis confirmed that sorafenib reduced protein and mRNA levels without altering PLAG1 knockdown and overexpression efficiency(Fig. 2A and B).

A key feature of ferroptosis is LPOs accumulation, often triggered by the depletion of GSH [28, 29]. We evaluated the viability of MHCC97H and MHCC97L cells, alongside GSH levels, to determine if PLAG1 could be a potential target for sorafenib-induced ferroptosis. Interestingly, PLAG1 knockdown significantly amplified sorafenib's inhibition of cell viability and depletion of GSH, in contrast to PLAG1 up-regulation (Fig. 2C and D). The levels of ferroptosis indicators including LPOs, Lipid ROS, and intracellular iron were assessed using specific probes. Results showed that sorafenib enhanced ferroptosis in MHCC97H cells, seen in elevated levels of intracellular iron, Liperfluo, and Lipid ROS. However, it suppressed ferroptosis in MHCC97L cells, with reduced levels of these markers (Fig. 2E-H).

Ferroptosis fundamentally occurs when a balance between oxidative damage and antioxidant defenses is lost, leading to rampant lipid peroxidation and compromising mitochondrial membrane integrity [30, 31]. This destabilization escalates to mitochondrial crest rupture, triggering ferroptosis. Transmission electron microscopy revealed that PLAG1 inhibition increases mitochondrial membrane density, decreases mitochondrial volume, and induces crest membrane disintegration on sorafenib treatment (Fig. 2I). Taken together, these results suggest that PLAG1 may serve as an attractive strategic target to boost ferroptosis in sorafenib's presence.

### PLAG1 controls the occurrence of sorafenib-induced ferroptosis through a PVT1-dependent mechanism with no change in protein stability

**Fig. 3:**
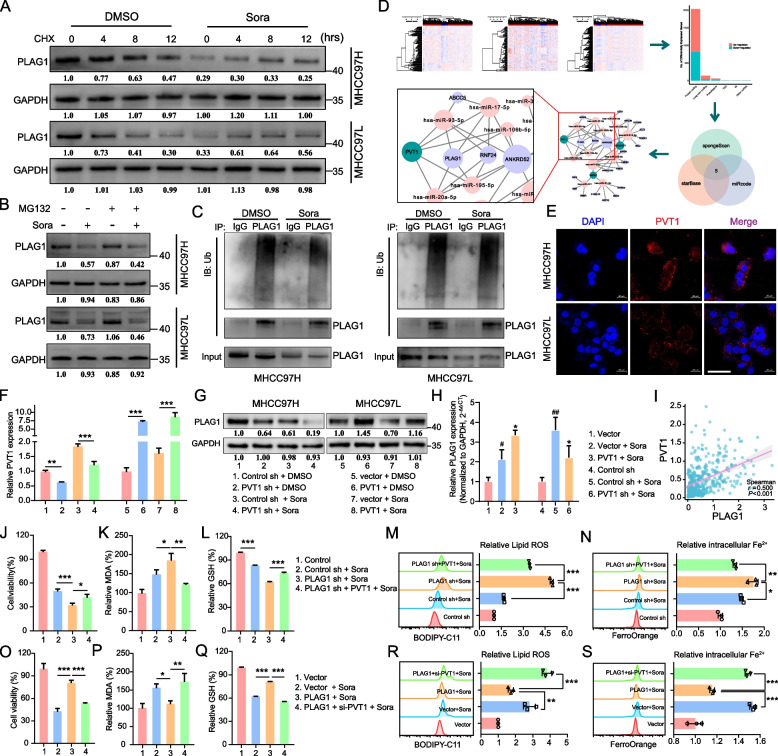
PLAG1 regulates lipid ROS, Fe^2+^ and MDA in a PVT1-dependent manner in HCC cells treated with sorafenib. The dependence of Sorafenib-induced PLAG1 expression on PVT1 was investigated in this study. (**A**-**B**) CHX and MG132 experiments showed that sorafenib induction had no effect on the protein stability of PLAG1. At the same time, PLAG1 ubiquitination experiments showed that the sorafenib experiment did not change the level of PLAG1 ubiquitination (**C**), and we speculated that post-transcriptional regulation may affect the difference in the expression trend of PLAG1 protein and mRNA. (**D**) The construction of the ceRNETs was illustrated in the flowchart. In this network, lncRNA is visually represented by the color green, mRNA by the color light purple, and miRNA by the color pink. Also we observed that PVT1 acts as a ceRNA for PLAG1. (**E**) The subcellular localization of PVT1 was detected in the cytosol using the FISH experiment, with a scale bar of 50 μm. MHCC97H and MHCC97L cells were transfected with indicator constructs and subsequently treated with either DMSO or sorafenib (10 μM) for a duration of 24 h. The mRNA levels of PVT1 were quantified using qRT-PCR (**F**), while the protein levels of PLAG1 were assessed through western blot analysis (**G**). qRT-PCR results showed that PVT1 was able to reverse the effect of sorafenib on PLAG1 at the mRNA level (**H**). Additionally, Spearman's correlation analysis was employed to determine the relationship between PVT1 mRNA levels and PLAG1 mRNA levels in patients with HCC (**I**). Enhancing the downregulation of PLAG1 promoted sorafenib-induced ferroptosis, while this effect was counteracted by upregulating PVT1. Genetically modified MHCC97H and MHCC97L cells were treated with sorafenib (10 μM) for a duration of 24 h. The viability of the cells was assessed using the CCK-8 kit (**J**), the formation of lipids was measured through the MDA assay (**K**), the concentration of GSH in the cells was determined using a glutathione assay kit (**L**), the accumulation of lipid ROS was analyzed by flow cytometry using BODIPY-C11 probes (**M**), and the concentration of intracellular Fe^2+^ was analyzed using FerroOrange probes (**N**). Downregulation of PVT1 rescued the inhibition of ferroptotic events caused by overexpression of PLAG1, which included reduction in cell viability (**O**), MDA levels (**P**), GSH depletion (**Q**), lipid ROS (**R**), and intracellular Fe^2+^ (**S**). The data displayed indicate mean ± SD obtained from three independent experiments. *P < 0.05; **P < 0.01; ***P < 0.001

Upon verification of the efficacy of PLAG1 knockdown and overexpression, it was observed that the protein and RNA levels of PLAG1 induced by sorafenib exhibited contrasting expression patterns. To elucidate this phenomenon, we initially investigated the impact of sorafenib on the stability of PLAG1 protein through CHX and MG132 experiments (Fig. 3A and B). The results showed that sorafenib treatment did not lead to a decrease in the protein stability of PLAG1. Furthermore, an investigation was conducted on the impact of sorafenib on PLAG1 ubiquitination, which resulted in the observation of no substantial changes in the ubiquitination status of PLAG1 after sorafenib administration((Fig. 3C).

ceRNAs are transcripts that interact with each other at the post-transcriptional level by competing for shared miRNAs [32]. These miRNAs, typically 21 ~ 23 nucleotides in length, guide the AGO protein to bind to target transcripts through base pairing, ultimately leading to either degradation or translational inhibition [33, 34]. Competitive endogenous RNA networks (ceRNETs) facilitate the interaction between protein-encoding mRNAs and non-coding RNAs, thereby potentially influencing post-transcriptional gene expression regulation in various biological states. Utilizing data from the TCGA database, we developed ceRNETs specific to hepatocellular carcinoma, revealing PVT1 as a potential ceRNA for PLAG1 through identification of central network nodes (Fig. 3D). In addition, our FISH analysis confirmed that PVT1 is mainly localized in the cytoplasm and the expression of PVT1 is upregulated in a concentration-dependent manner by sorafenib (Fig. 3E and and Supplementary Fig. 2).

The role of PVT1 in the pathogenesis of digestive system tumors, including promoting proliferation, metastasis, and autophagy of hepatoma cells, is well-established [35–37]. However, the impact of PVT1 on PLAG1 regulation remains unclear. We aimed to address this by using the MHCC97H/sh-PVT1 and MHCC97L/PVT1 cell lines in our study. Our qRT-PCR results indicated that sorafenib did not significantly affect PVT1 knockdown or overexpression efficacy, nor did it affect PVT1's positive regulation of PLAG1 as confirmed by western blotting (Fig. 3F and G). Further, we also found that PVT1 was able to reverse the effect of sorafenib on PLAG1 at the transcriptional level (Fig. 3H), and a positive correlation was identified between PVT1 and PLAG1 at the transcriptional level using Spearman's correlation analysis (Fig. 3I, *P* < 0.001, *r* = 0.500). These suggests that PVT1 may be an upstream factor regulating the upregulation of PLAG1 mRNA expression induced by sorafenib. And that's not all, we also found overexpression of PVT1 partially offset sorafenib-induced cell death due to PLAG1 deficiency in the MHCC97H cell line in a rescue experiment (Fig. 3J). Correspondingly, PVT1 overexpression mitigated sorafenib-induced MDA, lipid ROS, and Fe^2+^ production, whilst boosting GSH levels (Fig. 3K-N). Conversely, PVT1 inhibition increased ferroptotic processes, such as sorafenib-induced cell death and enhanced MDA, lipid ROS, and Fe^2+^ production, whilst reducing GSH levels (Fig. 3O-S). Overall, this indicates that PLAG1 suppresses sorafenib-induced ferroptosis through a PVT1-dependent mechanism.

### The interaction between miR-195-5p and PVT1 or PLAG1 takes place

**Fig. 4:**
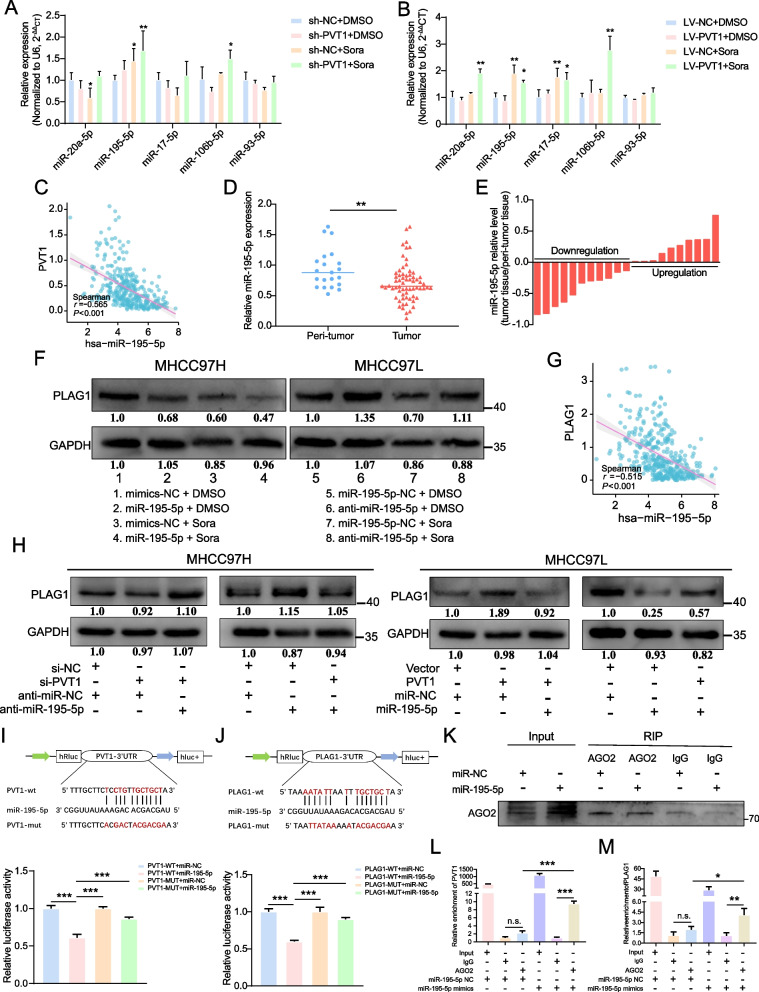
Both PVT1 and PLAG1 are interacted with miR-195-5p. The qRT-PCR examination of miR-20a-5p, miR-195-5p, miR-17-5p, miR-106b-5p, and miR-93-5p was conducted on MHCC97H cells (**A**) and MHCC97L cells (**B**) that were transfected with the indicator constructs and subsequently subjected to treatment with DMSO or sorafenib (10 μM) for a duration of 24 h. (**C**) Spearman's correlation investigation was performed to analyze the relationship between miR-195-5p expression and PVT1 expression using data from TCGA-LIHC. (**D**) Comparison of miR-195-5p expression levels in HCC and normal tissues using cDNA microarray analysis. (**E**) The levels of miR-195-5p mRNA expression in 21-paired tumor and peritumoral tissues obtained from patients with HCC were measured using the qRT-PCR method. The data were presented as − ΔΔCt values. (**F**) The impact of miR-195-5p on the protein level of PLAG1 was evaluated using western blot analysis, with the use of DMSO or sorafenib (10 μM) for a duration of 24 h. (**G**) Furthermore, a correlation analysis was conducted to investigate the association between the expression of miR-195-5p and PLAG1 in patients diagnosed with HCC. (**H**) Western blot analysis provided evidence of a reciprocal regulatory effect between PVT1 and miR-195-5p on the expression of PLAG1. The binding sites of miR-195-5p with PVT1 (**I**) or PLAG1 (**J**) were predicted using a public database. HEK293T cells were transfected with WT vectors or MUT vectors, along with miR-195-5p mimics and miR-NC, and luciferase activity was measured. (**K**) The expression of AGO2 protein was detected in MHCC97H cells using western blot analysis, while PVT1 (**L**) and PLAG1 (**M**) were detected using qRT-PCR. The data displayed indicate mean ± SD obtained from three independent experiments. *P < 0.05; **P < 0.01; ***P < 0.001; n.s., not significant

PVT1 has already been demonstrated to play a significant role in tumor growth via the ceRNA mechanism [38, 39]. By analyzing the constructed ceRNA network, we identified the specific miRNAs linked with PVT1. We observed miR-195-5p increased with PVT1 suppression in combination with sorafenib treatment, contrary to a drop in miR-195-5p levels when PVT1 was overexpressed (Fig. 4A and B). Further analysis of RNA-seq data from the TCGA database showed a negative correlation between miR-195-5p and PVT1 levels in 374 individuals with HCC (Fig. 4C, *P* < 0.001,* r* =  − 0.565). In addition to data analysis of TCGA-LIHC, we also discovered diminished miR-195-5p expression in tumor tissues compared to peri-tumor tissues through cDNA microarray (Fig. 4D, E and Supplementary Fig. 3A). The finding suggested a possible regulation by PVT1 of miR-195-5p controlling PLAG1.

Our subsequent findings revealed that the introduction of miR-195-5p mimics led to a decrease in PLAG1 protein levels in MHCC97H cells, while miR-195-5p inhibitors increased PLAG1 expression in MHCC97L cells. This regulatory pattern remained consistent during sorafenib treatment (Fig. 4F). Spearman’s correlation analysis showed a strong negative correlation between miR-195-5p and PLAG1 (Fig. 4G, *P* < 0.001, *r* =  − 0.515). Further, we found that PVT1 partially mitigates the suppressive effect of miR-195-5p on PLAG1 abundance and could partially reverse the enhancing effect of PVT1 on PLAG1 expression in HCC cells (Fig. 4H). These findings indicated that PLAG1 could be a target of the PVT1/miR-195-5p axis.

We then explored a direct interaction between miR-195-5p and PVT1 or PLAG1 messenger RNA. Interestingly, analysis indicated that miR-195-5p could bind to the 3' UTR region of both PVT1 and PLAG1 (Fig. 4I, J and Supplementary Fig. 3B and C). This information uncovers miR-195-5p as a downstream target of PVT1. The AGO2 protein, an integral component of the RNA-induced silencing complex, plays a pivotal role in facilitating the degradation of target mRNA or impeding its protein synthesis within the miRNA/siRNA pathway [40]. To ascertain the competition between PVT1 and PLAG1 in the binding of miR-195-5p within HCC cells, the anti-AGO2 RIP assay was employed to demonstrate AGO2's efficient capture by anti-AGO2 antibodies in MHCC97H cells transfected with miR-195-5p mimics (Fig. 4K), and suggested competition between PVT1 and PLAG1 for miR-195-5p binding within HCC cells. Increased levels of PVT1 and PLAG1 were then detected in the MHCC97H cell group transfected with miR-195-5p mimics compared to the control group, revealing that PVT1 may serve as a molecular sponge for miR-195-5p to regulate PLAG1 (Fig. 4L and M).

### MiR-195-5p reverses the impact of PLAG1 on ferroptosi.

**Fig. 5:**
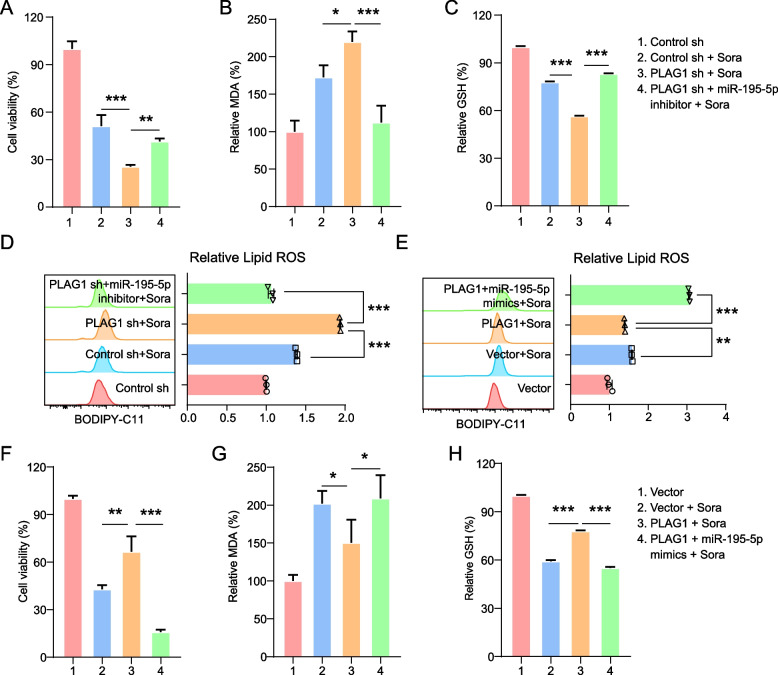
The inhibition of ferroptosis is observed through the regulation of PLAG1 by miR-195-5p. The inhibition of PLAG1 resulted in an augmentation of sorafenib-induced ferroptosis, which was counteracted by the miR-195-5p inhibitor. Cell viability was assessed using the CCK-8 kit (**A**) following sorafenib treatment in MHCC97H cells transfected with the construct. Lipid formation was measured using the MDA assay (**B**), GSH concentration was measured using the glutathione assay kit (C), and lipid ROS accumulation was measured using BODIPY-C11 probes (**D**). In MHCC97L cells treated with sorafenib (10 μM) for 24 h, the overexpression of PLAG1 suppressed ferroptotic events, such as lipid ROS (E), cell viability (F), MDA levels (G), and GSH depletion (H), while the introduction of miR-195-5p mimics rescued these effects. The data presented represent the mean ± SD obtained from three independent experiments. Statistical significance was determined using *P < 0.05, **P < 0.01, and ***P < 0.001

In rescue experiments using miR-195-5p inhibitors and mimics, we found that miR-195-5p inhibitor use led to a decrease in sorafenib-induced cell death in MHCC97H/sh-PLAG1 cells and also inhibited the accumulation of MDA and lipid ROS triggered by PLAG1 depletion (Fig. 5A, B and D). Additionally, miR-195-5p suppression intensified the drop in GSH levels following PLAG1 knockdown (Fig. 5C). A contrast was observed with the upregulation of PLAG1 in MHCC97L cells which countered the sorafenib-induced ferroptotic events suppressed by miR-195-5p overexpression (Fig. 5E-H). Such data indicate that PLAG1's influence on sorafenib-induced ferroptosis is regulated by miR-195-5p levels.

### GPX4 functions as the downstream effector of PLAG1 to influence ferroptosis inhibition

**Fig. 6:**
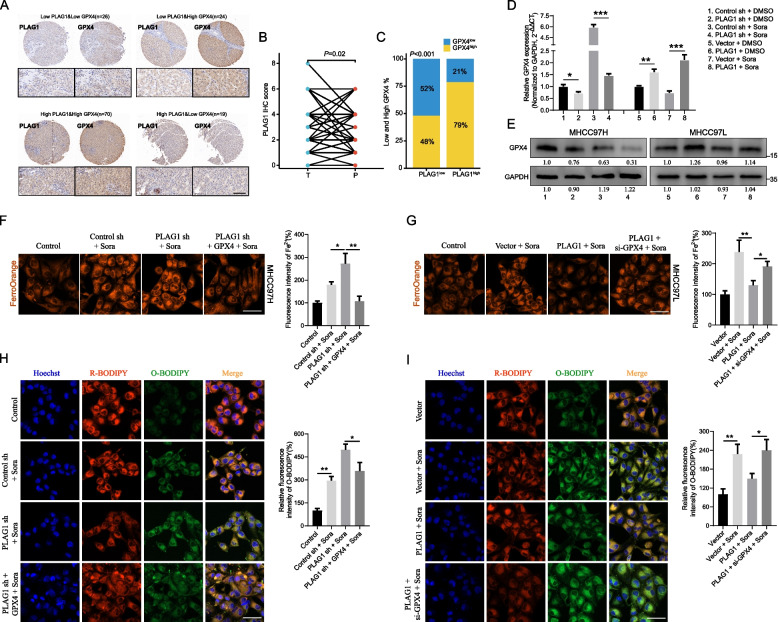
GPX4 functions as the downstream target of PLAG1. The expression of PLAG1 shows a positive correlation with the expression of GPX4 in HCC tissues. (A) Representative figures of the expression patterns of PLAG1 and GPX4 in the indicated groups. Scale bar: 100 μm. (B) IHC score of PLAG1 in 139 HCC samples and peritumoral samples were compared. (C) The percentages of tumor tissues with high or low levels of GPX4 expression in individuals with high or low levels of PLAG1 expression among a total of 139 HCC samples. In MHCC97H treated with either DMSO or sorafenib (10 µM) for 24 h, the protein and mRNA expression levels of GPX4 were reduced by the knockdown of PLAG1. Conversely, the overexpression of PLAG1 in MHCC97L led to an increase in both the mRNA and protein expression levels of GPX4 (D, E). The intracellular Fe^2+^ was detected by the FerroOrange probe, scale bar = 50 μm (F, G). And lipid ROS accumulation was analyzed by C11-BODIPY^581/591^ probe, scale bar = 50 μm (H, I). The data presented represent the mean ± SD obtained from three independent experiments. Statistical significance was determined using *P < 0.05, **P < 0.01, and ***P < 0.001

The GSEA enrichment analysis demonstrated that the co-expression of PLAG1 with differentially expressed genes is associated with a regulatory role in the oxidation–reduction reaction (Supplementary Fig. 4A). Our study investigated the role of PLAG1 in enhancing GPX4 expression in HCC, given GPX4's regulatory function in cellular antioxidant responses. Initially, we examined the correlation between PLAG1 and GPX4 in tissues obtained from HCC patients. In order to establish a correlation between PLAG1 and GPX4, IHC analysis was performed on a total of 139 samples of tumor and peritumoral tissues obtained from the HCC patient cohort. The distribution of PLAG1 and GPX4 was observed in both the cytoplasm and nucleus of HCC cells and peritumoral hepatocytes. The expression patterns of PLAG1 and GPX4 in the tumor tissues of the four groups were depicted in Fig. 6A, with a score of four or higher indicating positive or high expression, in conjunction with the intensity and diversity of IHC staining. According to the IHC scoring, the levels of PLAG1 in tumor tissues were significantly higher compared to those in peritumoral tissues (Fig. 6B). Interestingly, 52% of tumor tissue samples with decreased PLAG1 levels also showed reduced GPX4 levels, whereas 79% of samples with increased PLAG1 levels showed elevated GPX4 expression (Fig. 6C). This finding strongly suggests a direct association between the two in HCC tissues and is backed by the TCGA database analysis which depicted a positive correlation between PLAG1 mRNA and GPX4 mRNA (Supplementary Fig. 4B). Further experiments in liver cancer cells validated this, showing a significant decrease in GPX4 levels in MHCC97H/shPLAG1 cells when compared to the control group. Conversely, an upsurge in these levels was observed in MHCC97L/PLAG1 cells (Fig. 6D and E). It appears that the rise in GPX4 protein in relation to PLAG1 expression could be due to transcriptional regulatory mechanisms.

Lipid ROS and Fe^2+^ are two important downstream indicators of GPX4 inhibition of ferroptosis. We further assessed their level in sorafenib-treated MHCC97H/sh-PLAG1 and MHCC97L/PLAG1 through FerroOrange and C11 BODIPY^581/591^ probe. We found that overexpression of GPX4 could reverse the upregulation of Fe^2+^ and lipid ROS promoted by PLAG1 knockdown, while GPX4 interference rescued the downregulation of Fe^2+^ and lipid ROS caused by overexpression of PLAG1 (Fig. 6F-I). In short, our results verified the crosstalk between PLAG1 and GPX4 in ferroptosis inhibition.

### The mapping of chromatin state identifies the presence of PLAG1 in the promoter region of GPX4

**Fig. 7:**
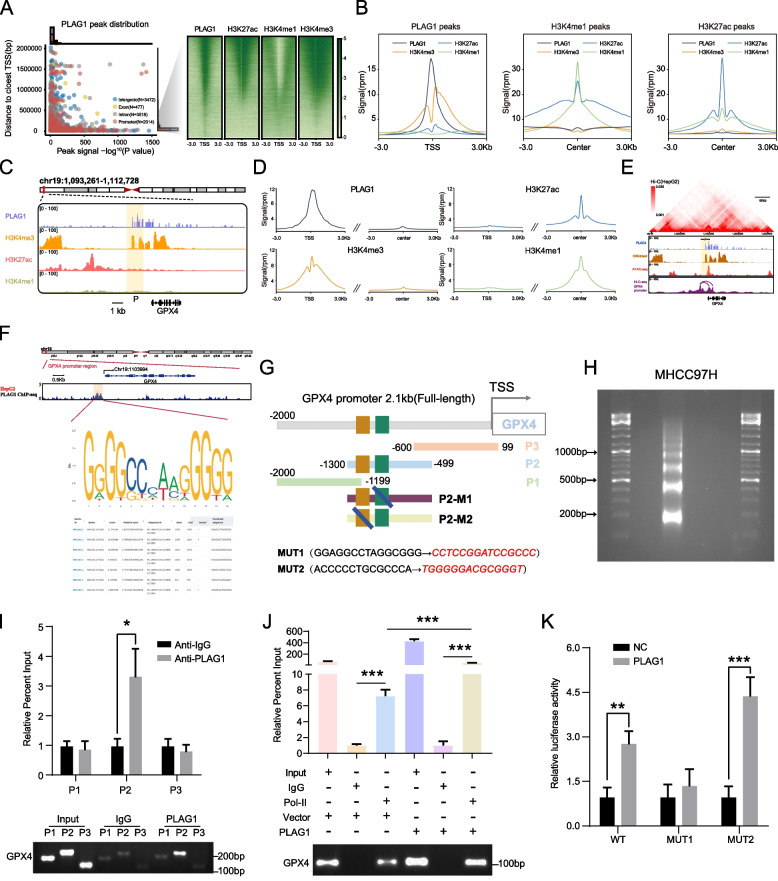
The mapping of chromatin state identifies the presence of PLAG1 in the promoter region of GPX4. (**A**) The distribution of PLAG1 peaks and the heatmaps displaying PLAG1, H3K27ac, H3K4me1, and H3K4me3 at regulatory elements in close proximity to HepG2 cells are depicted on the left and right sides, respectively. (**B**) The signals of PLAG1, H3K27me3, H3K27ac, and H3K27me1 at the peaks of PLAG1, H3K27me1, and H3K27ac are illustrated on the left, middle, and right sides, respectively. (**C**) Additionally, gene traces for PLAG1, H3K4me3, H3K27Ac, and H3K4me1 ChIP-seq presence at the promoter of GPX4 in HepG2 cells are shown. The ChIP-seq coverage is expressed as reads per million per base pair (rpm/bp). (**D**) The metagene analysis illustrates the global presence of PLAG1, H3K4me3, H3K27ac, and H3K4me1 at the promoters encompassing the transcription start site (TSS) and enhancer regions. (**E**) The presence of PLAG1, H3K4me3, H3K27ac ChIP-seq data, and ATAC-seq data in the vicinity of the GPX4 locus was examined in HepG2 cells, revealing a prominent Hi-C profile. Chromatin looping in HepG2 cells brings the distal GPX4 region closer to its target promoter, as indicated by the arrows. (**F**) The binding motif of PLAG1 and the top seven binding sites in the GPX4 promoter were identified using JASPAR online tools. (**G**) A diagram is presented to illustrate the truncation pattern based on the spatial distribution of the binding sites. (**H**) The results obtained from DNA gel electrophoresis indicate that the chromatin fragments treated with micrococcal nuclease were predominantly observed within the range of 200 to 500 bp. (**I**) In order to investigate the binding of PLAG1 to the P2 truncator of the GPX4 promoter region (-2000 bp ~  + 99 bp) in MHCC97H, ChIP assays and qRT-PCR were conducted. The levels of truncated genomic DNA containing the predicted binding sequence in MHCC97H cells were visualized using DNA gel electrophoresis. (**J**) In order to determine the strength of RNA polymerase II (Pol II) binding to the central region of the GPX4 promoter (-250 bp to 120 bp) in MHCC97H, ChIP assays and qRT-PCR were performed. The presence of gDNA containing the core region of the GPX4 promoter in MHCC97H cells was visualized using DNA gel electrophoresis. (**K**) To investigate the specific binding sequence between PLAG1 and the P2 truncator of the GPX4 promoter, mutant groups were constructed based on the predicted results of the JASPAR database and the luciferase reporter assay was utilized to identify changes in luciferase intensity relative to controls in the case of PLAG1 overexpression. The data presented represent the mean ± SD obtained from three independent experiments. Statistical significance was determined using *P < 0.05, **P < 0.01, and ***P < 0.001

We undertook a reanalysis of ChIP-seq data to understand the mechanism through which PLAG1 maintains GPX4 gene expression and discovered that around 25% selected by PLAG1 were within 3 kb of the transcription start site (TSS) (Fig. 7A and Supplementary Fig. 5A-C). H3K4me3 is mainly enriched in the promoter region near the TSS, while most H3K4me1 and H3K27ac modifications are enriched in the enhancer region [41]. All identified TSS had the H3K4me3 marker, associated with active promoters, but lacked active enhancer markers H3K27ac and H3K4me1(Fig. 7A and B). However, regions marked with H3K4me1 didn't have PLAG1 protein and H3K4me3** (**Fig. 7B**)**. Through a comprehensive analysis of the enrichment patterns of PLAG1, H3K4me3, H3K4me1, and H3K27ac in the 3 kb region flanking the TSS of GPX4, it is hypothesized that the transcription factor PLAG1 interacts with the GPX4 promoter to enhance GPX4 transcription.

In eukaryotic cells, the formation of chromatin complexes can result in tightly folded structures, allowing for potential interactions between distant regions on the DNA sequence [42]. This interaction increases the likelihood of enhancers binding to transcription factors and RNA Pol II. Figure 7C and D revealed the co-occupation of PLAG1 and H3K4me3 in the promoter region of GPX4. Furthermore, the Hi-C profile suggested that the enhancers of GPX4 may interact with the promoter via a "loop" mechanism, aiding transcription activation (Fig. 7E).

The Jaspar database was employed to study the potential direct interaction between PLAG1 and GPX4 promoter elements, revealing PLAG1's key binding patterns' ability to interact with the GPX4 promoter (Fig. 7F and G). Crosslinked chromatin fragments were treated with nucleic acid micrococcal nuclease and sonication techniques resulting in chromatin fragments from 200 to 500 bp in size (Fig. 7H). The ChIP assay strongly associates PLAG1 with the P2 region (-1300 to -499 bp) of GPX4 promoter (Fig. 7I). Additionally, a significant enrichment was observed in the Pol II group within the GPX4's central promoter region, suggesting GPX4 transcription activation (Fig. 7J). When co-transfected with P2 region reporter plasmids in MHCC97H/PLAG1 cells, there was a significant increase of luciferase activity in the WT and MUT2 groups (Fig. 7K), suggesting the region between -933 bp and -919 bp as PLAG1's confirmed binding site. This evidence firmly links PLAG1's role in GPX4 gene expression.

### *The effectiveness of sorafenib is enhanced by inhibiting PLAG1 *in vivo

**Fig. 8:**
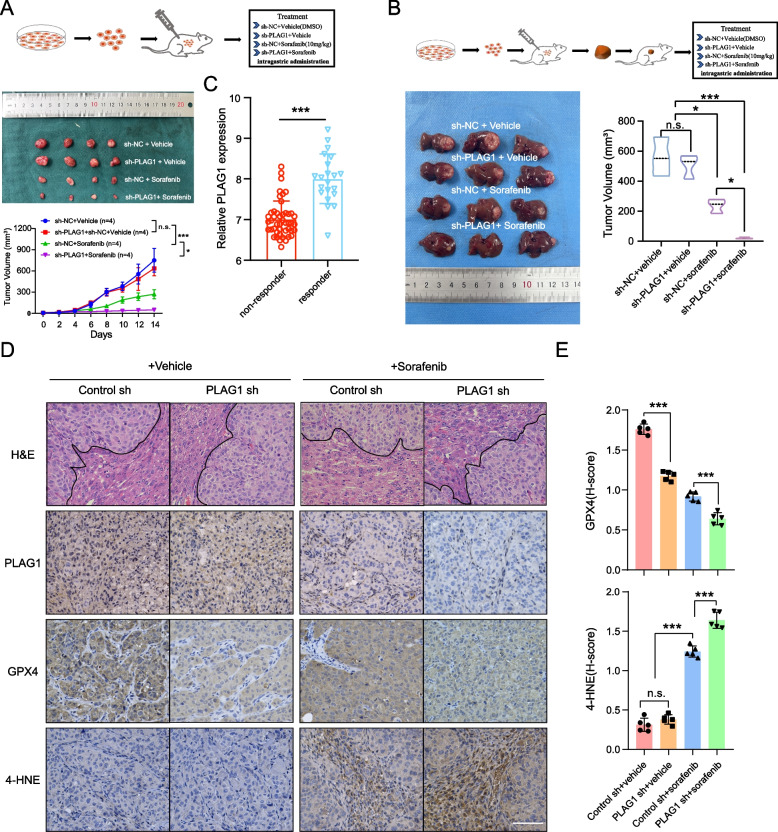
The effectiveness of sorafenib in vivo is enhanced by inhibiting PLAG1. (**A**) MHCC97H cells were cultured and subsequently injected subcutaneously into 4-week-old BALB/c nude mice. Once the mice's tumor volume reached 50 mm^3^, sorafenib was administered intragastrically at a dosage of 10 mg/kg per day. The tumor size was measured every other day until the 14th day, at which point the mice were sacrificed. (**B**) BALB/c nude mice were implanted with 5 mm^3^ tumor tissue derived from either MHCC97H/sh-NC or MHCC97H/sh-PLAG1 cells in situ. After one week of implantation, the respective groups were administered sorafenib (10 mg/kg, once daily) via intragastric administration. After 21 days of implantation, the mice were euthanized, and their livers were dissected to measure tumor volumes. (**C**) External data from GSE109211 examined the response of PLAG1 mRNA levels to sorafenib. (**D**) The provided representative IHC images of PLAG1, GPX4, and 4-HNE in tumor tissues from the specified orthotopic models were shown. The scale bar represents 100 μm. (**E**) The statistical results of GPX4 and 4-HNE were presented, indicating mean ± SD obtained from three independent trials. *P < 0.05; **P < 0.01; ***P < 0.001; n.s., not significant

The effectiveness of Sorafenib in inhibiting tumor growth was assessed in subcutaneous and orthotopic mouse models, in conjunction with PLAG1 level reduction. Our results showed that sorafenib had a higher efficacy in reducing tumor volume in MHCC97H/sh-PLAG1 cells than the control group (Fig. 8A and B). The mRNA levels of PLAG1 were analyzed in 21 sorafenib responders and 46 sorafenib non-responders with HCC from the GSE109211 dataset. The findings revealed that the mRNA levels of PLAG1 were significantly elevated in sorafenib responders compared to sorafenib non-responders (Fig. 8C). This observation aligns with our prior in vitro investigations, wherein treatment of HCC cells with sorafenib led to an increase in PLAG1 mRNA expression.

Additionally, reduced GPX4 expression levels were observed in tumors derived from MHCC97H/sh-PLAG1 cells compared to the control. Moreover, sorafenib also significantly reduced GPX4 signals in tumor tissues compared to vehicle-treated groups (Fig. 8D and E). To investigate potential ferroptosis, we stained the experimental tumor tissue with 4-HNE, a known ferroptosis indicator. The result showed increased 4-HNE levels in tumors from MHCC97H/sh-PLAG1 cells treated with sorafenib, in comparison to the control group (Fig. 8D and E). These findings suggest that PLAG1 inhibition can amplify the effectiveness of sorafenib in treating animal tumors via GPX4 downregulation and ferroptosis promotion.

## Discussion

Addressing the high mortality rate and poor prognosis associated with liver cancer poses considerable challenges, primarily due to limited treatment options for advanced disease. It underlines the urgent need for identifying new therapeutic targets. Our study indicates PLAG1 as a potential therapeutic target, given its increased expression levels in liver cancer tissues compared to normal ones. Utilizing the TCGA database, we found a correlation between high PLAG1 expression and reduced survival rates among liver cancer patients. While the drug sorafenib has revolutionized the treatment of advanced liver cancer, its efficacy is hampered by tumor heterogeneity and acquired drug resistance. Interestingly, our study observed that PLAG1 expression decreases with increasing dosages of sorafenib. Additionally, PLAG1 overexpression hinders the therapeutic effects of sorafenib on hepatoma cells. These results highlight the critical role of PLAG1 in sorafenib's therapeutic efficacy.

Ferroptosis is an iron-dependent form of cell death that has been linked to various metabolic and homeostasis disorders [30]. Recent studies show its role in the progression of several diseases including stroke, autoimmune disorders, and cancers such as renal, leukemia, pulmonary, and liver carcinoma [43]. This discovery presents a promising strategy in cancer research, encouraging the demise of cancer cells through ferroptosis. One particular observation is that sorafenib, a known cancer drug, can trigger ferroptosis in liver cancer treatment by blocking cystine intake which reduces GSH levels and causes accumulation of lipid ROS—ultimately inducing ferroptosis [44]. Previous investigations have provided evidence that sorafenib possesses the capability to induce the onset of ferroptosis in the treatment of liver cancer [6, 7], thus providing the possibility of confirming the inverse regulatory relationship between PLAG1 and ferroptosis. In this investigation, it was ascertained that the suppression of PLAG1, an oncoprotein, enhances the effects of sorafenib. More specifically, MHCC97H/sh-PLAG1 cells exhibited increased lipid peroxidation, decreased intracellular GSH, increased cellular contractions, and an elevation in mitochondrial membrane density. In addition, we also found that PLAG1 exhibited higher knockdown/overexpression efficiency in the sorafenib-induced group compared with the DMSO group. This not only makes up for the shortcomings of the relatively low knockdown/overexpression efficiency of the DMSO group, but also provides favorable support for the later study of sorafenib related ferroptotic events. In short, this suggests that PLAG1 plays a significant role in regulating cellular death and could provide insight into new cancer treatment strategies.

Ferroptosis has been closely linked to non-coding RNA (ncRNA) and cancer [45]. These ncRNAs are implicated in the fundamental regulatory processes of ferroptosis, encompassing mitochondria-associated proteins, iron metabolism, glutathione metabolism, and lipid peroxidation [46, 47]. Recent research has increasingly demonstrated the significant involvement of ncRNAs in the biological functionality of ceRNETs in liver cancer, influencing the binding affinity of miRNAs to target RNAs through the sharing of miRNA response elements [32]. PVT1, a major oncogenic long non-coding RNA in the gastrointestinal tract, is known to regulate the progression and drug resistance of hepatocellular carcinoma. It modulates downstream genes and biological pathways, impacting various human diseases, including cancer. In particular, PVT1 has been found to control TFR1 and p53 through its function as a miR-214 sponge, suppressing ferroptosis in cases of cerebral ischemia–reperfusion injury [48]. This was a first-time discovery linking PVT1 to ferroptosis. Later, research by He et al. [49] found that PVT1 interacts directly with miR-214-3p, preventing it from inhibiting GPX4. However, our understanding of PVT1's role in ferroptosis remains incomplete given the divergent phenotypic outcomes observed. Our study revealed that PVT1 counteracts the suppressive effect of sorafenib on PLAG1, and it enhances PLAG1 expression through its interaction with miR-195-5p. This underlines PLAG1's central role in the regulatory network.

GPX4 is a potential biomarker for ferroptosis, playing a key role in handling LPO levels [50, 51]. Its inactivation can imbalance oxidative processes, disturb membrane structures, and trigger ferroptosis, making GPX4 an attractive therapeutic target. Our GSEA enrichment analysis on the TCGA database indicates a strong connection between PLAG1 co-expression genes and redox reactions, suggesting GPX4 could be an influential mediator of PLAG1 in liver cancer. Simultaneously, our observations revealed that sorafenib induced a decrease in GPX4 protein levels, diverging from its RNA expression pattern. Consistent with findings by He et al. [49], our rescue experiments demonstrated that PVT1 can counteract the up-regulation of GPX4 mRNA levels (Supplementary Fig. 6), suggesting a regulatory influence of PVT1 on both PLAG1 and GPX4 at the upstream level.

Previous studies have observed decreased methylation in the GPX4 promoter region and increased levels of H3K4me3 and H3K27ac upstream of GPX4 in various tumor cells [52], indicating a potential link between heightened GPX4 expression, decreased methylation, and increased histone acetylation in tumors. Our follow-up study found that PLAG1 interacts with the GPX4 promoter and enhances its transcriptional activity in the MHCC97H cell line. Using a truncation assay and analyzing dual-luciferase reporter gene results, we identified region P2/ − 1300 bp ~  − 499 bp as the main area for PLAG1 and GPX4 promoter interaction based on its highest predicted binding score in the JASPRA database.

**Fig. 9:**
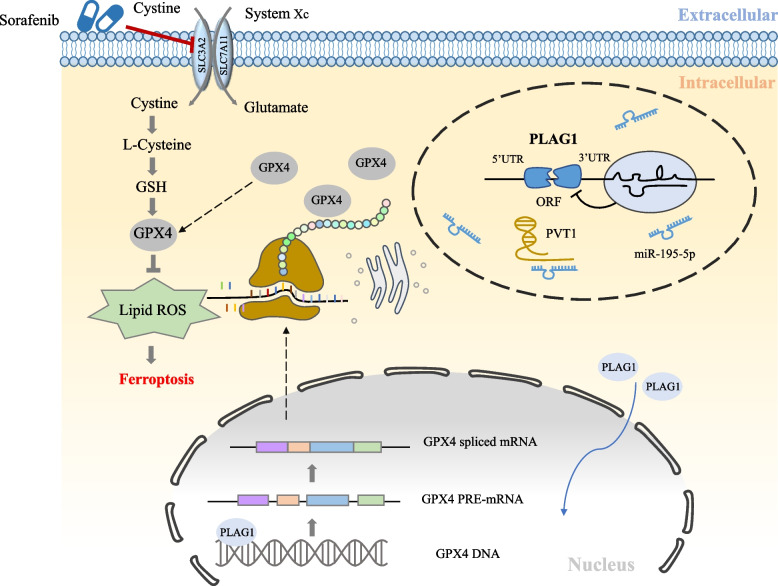
Schematic diagram of the present study. In HCC cells, PVT1 acts as a sponge for miR-195-5p, thereby upregulating the expression of PLAG1. Consequently, the increased levels of PLAG1 enhance the expression of GPX4, leading to the inhibition of the ferroptosis signaling pathway

In summary, our research has clarified the role of PLAG1 in sorafenib-induced ferroptosis. We discovered that PLAG1 downregulation enhances ferroptosis through the regulation of the PVT1/miR-195-5p axis. Additionally, the transcription factor PLAG1, stimulated by upstream factors, interacts with and promotes the transcription of target GPX4, affecting cell death induced by sorafenib treatment in both in vivo and in vitro models (Fig. 9). These findings highlight that PLAG1 interacts with GPX4 to conquer vulnerability to sorafenib induced ferroptosis through a PVT1/miR-195-5p axis-dependent manner and reinforce the potential of PLAG1 as a therapeutic target for sorafenib in the treatment of HCC.

### Supplementary Information

Supplementary Material 1.

## Data Availability

RNA-seq data were deposited into the GEO database (https://www.ncbi.nlm.nih.gov/geo) under accession number GSE182185 and GSE109211. The Hi-C and ChIP-seq data were also downloaded from GEO datasets GSE184796 and GSE151287. TCGA-LIHC cohort are available at the following URL: https://portal.gdc.cancer.gov/.

## Abbreviations

HCC: Hepatocellular carcinoma
RCD: Regulated cell death
PLAG1: Pleiomorphic adenoma gene 1
GPX4: Glutathione peroxidase 4
LPOs: Lipid peroxides
PVT1: Plasmacytoma variant translocation 1
HIF1: Hypoxia-inducible factor 1
GSH: Glutathione
qRT‑PCR: Quantitative real‑time polymerase chain reaction
IHC: Immunohistochemistry
H-score: Histological score
HRP: Horseradish peroxidase
DMSO: Dimethyl sulfoxide
MDA: Malondialdehyde
TBA: Thiobarbituric acid
ROS: Reactive oxygen species
NC: Normal control
Pol II: Polymerase II
ChIP: Chromatin immunoprecipitation
ceRNA: Competing endogenous RNA
WT: Wild type
MUT: Mutant type
RIP: RNA immunoprecipitation
FISH: Fluorescence in situ hybridization
TADs: Topologically associated domains
TSS: Transcription start site
