# Supplementary material for: PLAG1 interacts with GPX4 to conquer vulnerability to sorafenib induced ferroptosis through a PVT1/miR-195-5p axis-dependent manner in hepatocellular carcinoma
Source: J Exp Clin Cancer Res. 2024 May 14;43:143. doi: 10.1186/s13046-024-03061-4 (PMC11092053; doi:10.1186/s13046-024-03061-4)
Supplement: Supplementary file 1 — Supplementary Material 1. [file 13046_2024_3061_MOESM1_ESM.docx]

**Supplementary information for**

PLAG1 interacts with GPX4 to conquer vulnerability to sorafenib induced ferroptosis through a PVT1/miR-195-5p axis-dependent manner in hepatocellular carcinoma

*Jiarui Li,^a^ Yilan Li ,^a^ Denghui Wang,^a^ Rui Liao,^a^ and Zhongjun Wu^a,^**

**This file includes:**

Supplementary figures 1 to 6

Supplementary figure legends 1 to 6

Supplementary tables 1 to 3


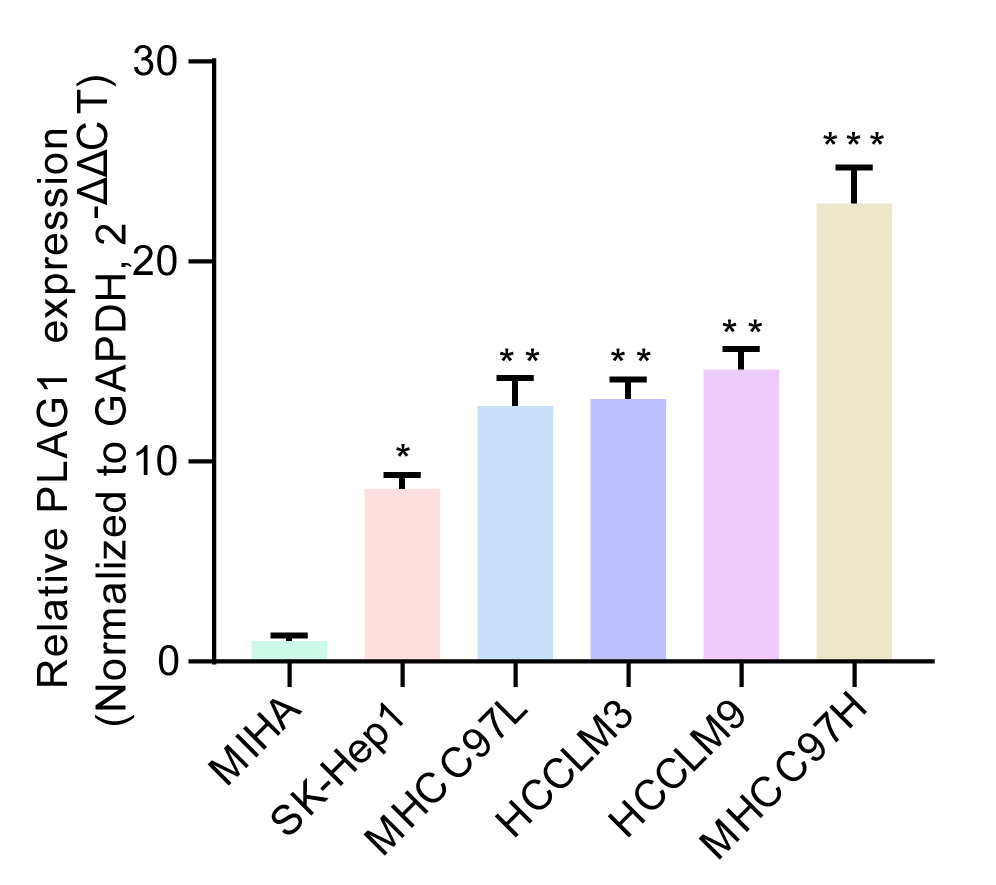


**Supplementary Fig. 1** The increased expression of PLAG1 in five distinct human HCC cell lines versus the hepatocyte MIHA cell line. Data shown represent mean ± SD from three independent experiments. * *P* < 0.05; ***P* < 0.01; ****P* < 0.001;


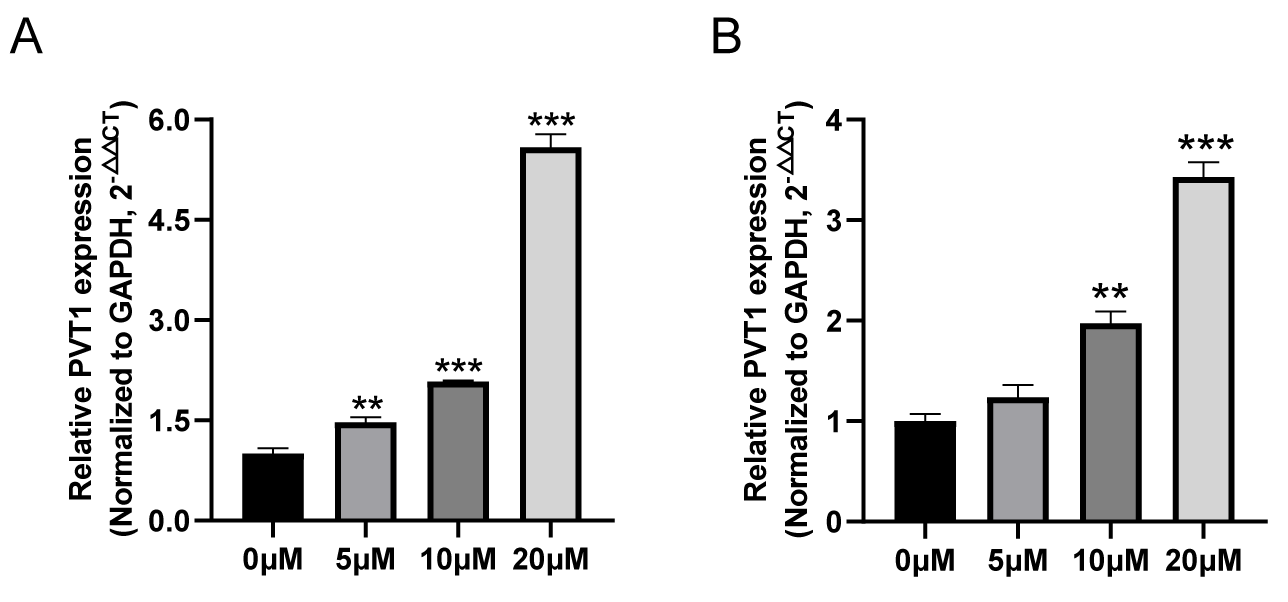


**Supplementary Fig. 2** The expression level of PVT1 increased in a concentration-dependent manner with sorafenib. Data shown represent mean ± SD from three independent experiments. **P* < 0.05; ***P* < 0.01; ****P* < 0.001;


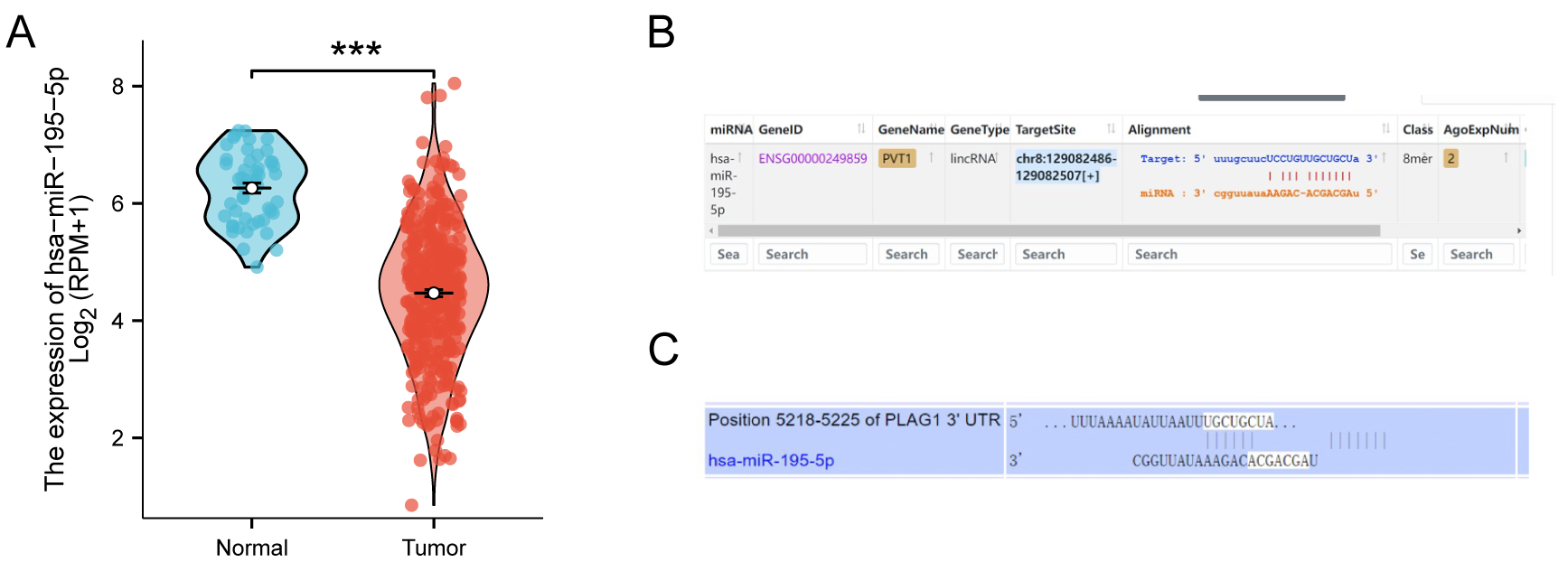


**Supplementary Fig. 3 The interaction between miR-195-5p and PVT1 or PLAG1 takes place.(A)** miR-195-5p expression is reduced in tumor tissues compared to peritumor tissues. Detailed information on the 3' UTR predicted by the starBase database for miR-195-5p in combination with PVT1 **(B)** and PLAG1 **(C)**.


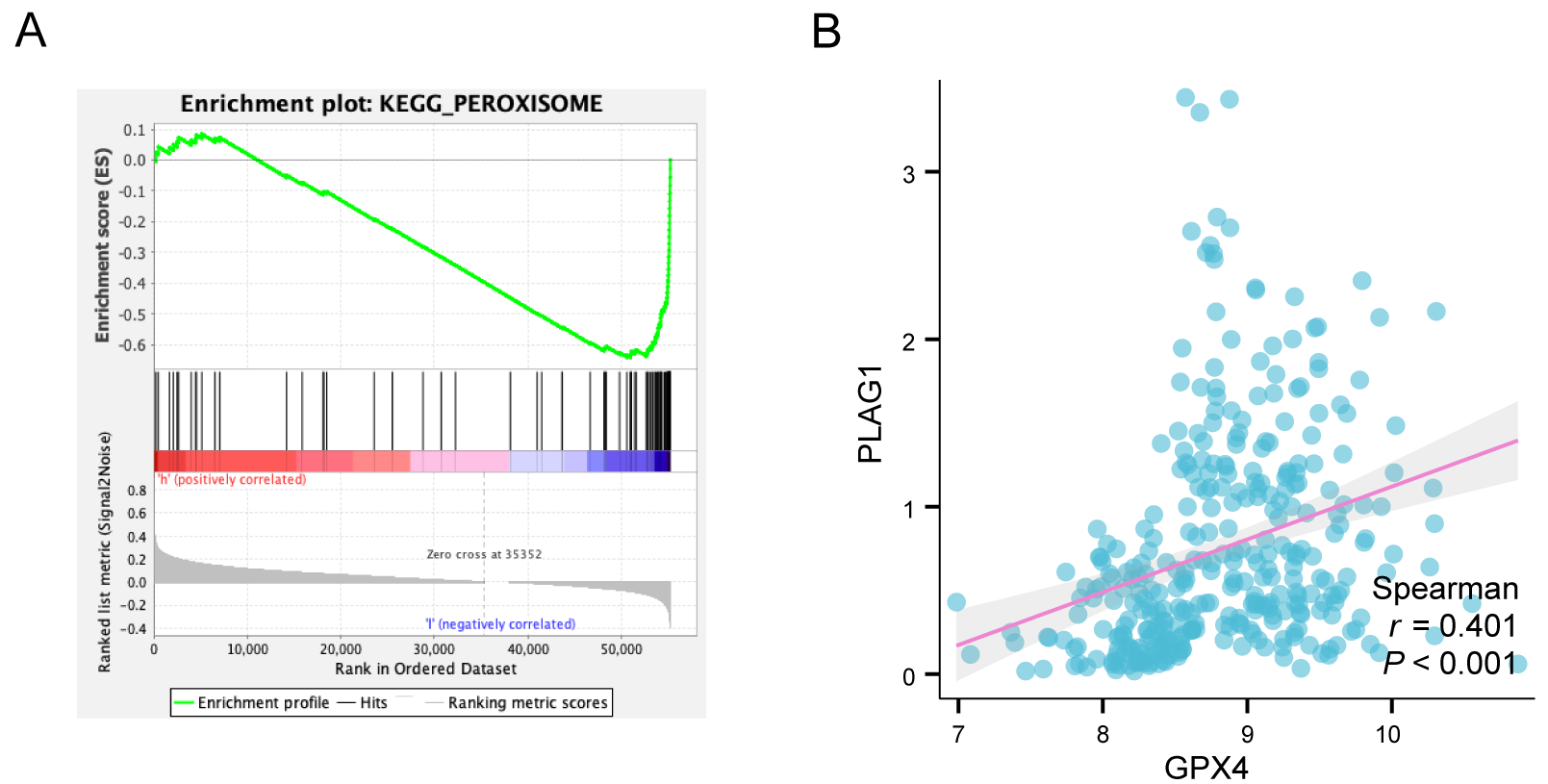


**Supplementary Fig. 4 GPX4 functions as the downstream effector of PLAG1. (A)** The GSEA enrichment analysis demonstrated that the co-expression of PLAG1 with differentially expressed genes is associated with a regulatory role in the oxidation-reduction reaction. **(B)** TCGA database analysis depicted a positive correlation between PLAG1 mRNA and GPX4 mRNA.


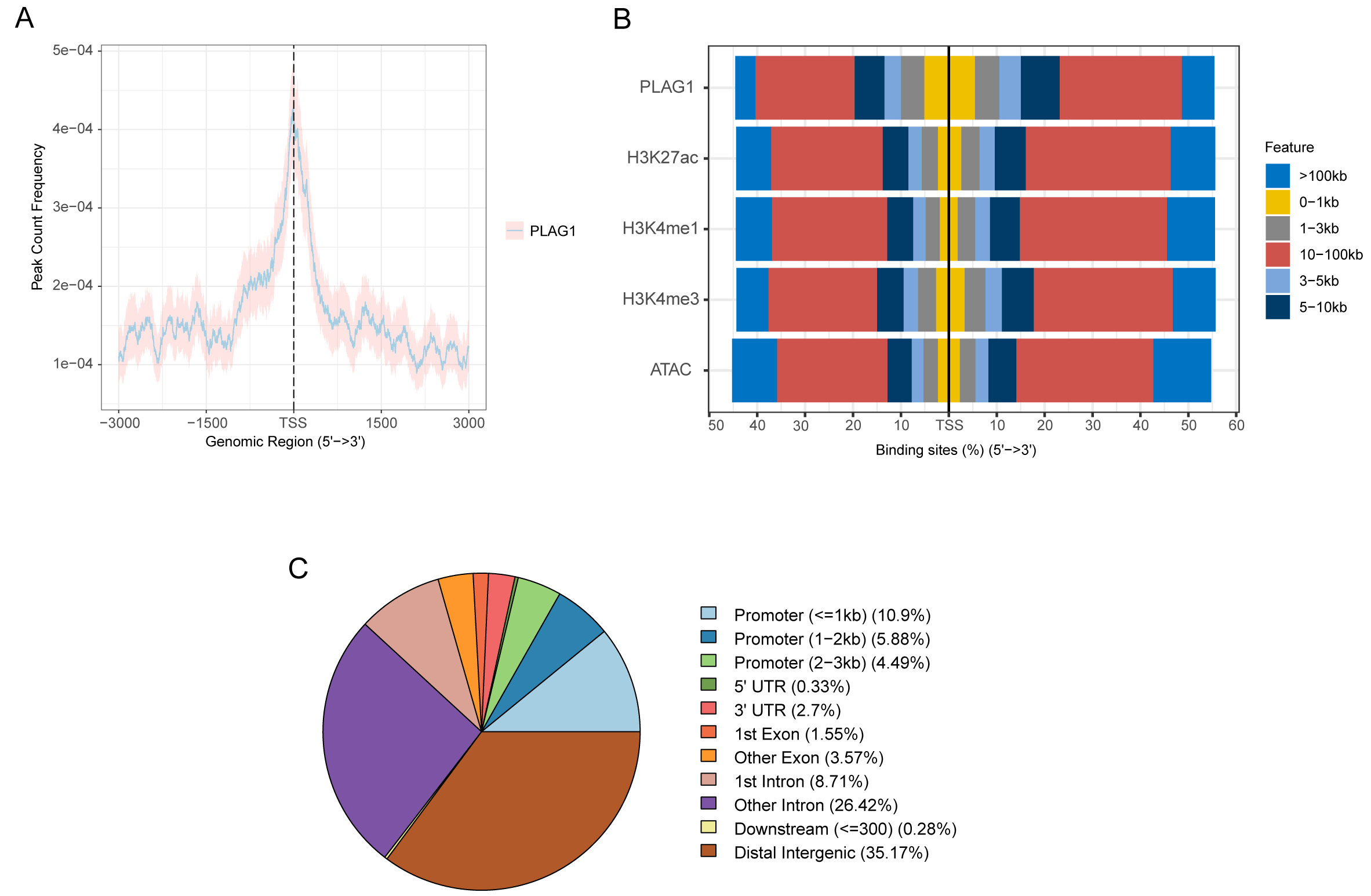


**Supplementary Fig. 5 The mapping of chromatin state identifies the presence of PLAG1 in the promoter region of GPX4. (A)** Average profile of ChIP peaks binding to TSS region. **(B)** Distribution of PLAG1, H3K27ac, H3K4me1 and H3K4me3 binding loci relative to TSS. **(C)** Genomic annotation of PLAG1 by pieplot.

**Supplementary Fig. 6** qRT-PCR experiments confirmed that knockdown of PVT1 could counteract sorafenib-induced upregulation of GPX4 mRNA levels. ^##^*P* < 0.01 and ^###^*P* < 0.001 vs Vector group or Control sh group; * *P* < 0.05 vs Vector + Sora group or Control sh + Sora group;

**Supplementary Table 1**

Primer sequences of qRT-PCR

|  |  |  | **Primer sequences** |  |
| --- | --- | --- | --- | --- |
| PLAG1-Forward  PLAG1-Reverse  PVT1-Forward  PVT1- Reverse  GPX4-Forward  GPX4-Reverse  GAPDH-Forward  GAPDH-Reverse |  |  | 5'-TCAAATATCCGTTCAGTTCTACCTC-3'  5'-CCTAGCTTAGATGATGACGATGC-3'  5'-CATAGATCCTGCCCTGTTTGC-3'  5'-GCAGTAGCCTCAGTGAACTCCTC-3'  5'-TTTCCGCCAAGGACATCG-3'  5'-TTACTTCGGTCTTGCCTCACTG-3'  5'-CACTCCTCCACCTTTGACGC-3'  5'-CTGTTGCTGTAGCCAAATTCGT-3' |  |

| hsa-miR-20a-5p | F primer | GCCGTAAAGTGCTTATAGTGCAG |
| --- | --- | --- |
|  | R primer | TATGGTTTTGACGACTGTGTGAT |
|  | **Size: 70bp** | |
| U6 | F primer | CAGCACATATACTAAAATTGGAACG |
|  | R primer | ACGAATTTGCGTGTCATCC |
|  | **Size:76bp** | |
| hsa-miR-17-5p | F primer | ATTCTTCCAAAGTGCTTACAGTGC |
|  | R primer | TATGGTTTTGACGACTGTGTGAT |
|  | **Size:70bp** | |
| hsa-miR-195-5p | F primer | CGTTATCCTAGCAGCACAGAAAT |
|  | R primer | TATGGTTTTGACGACTGTGTGAT |
|  | **Size:66bp** | |
| hsa-miR-93-5p | F primer | CGTTATATCCCAAAGTGCTGTTC |
|  | R primer | TATGGTTGTTCTCGTCTCCTTCTC |
|  | **Size:72bp** | |
| hsa-miR-106b-5p | F primer | AAATGCTCATAAAGTGCTGACAGT |
|  | R primer | TATGGTTTTGACGACTGTGTGAT |
|  | **Size:66bp** | |

**Supplementary Table 2**

Primer sequences of ChIP-PCR

|  |  | |  | **Primer sequences** |
| --- | --- | --- | --- | --- |
| GPX4  (anti-PLAG1)  GPX4  (anti-Pol-II) | | P1-Forward  P1-Reverse  P2-Forward  P2-Reverse  P3-Forward  P3-Reverse  Forward  Reverse |  | 5'-TCACTGCCTCTGCCACCAA-3'  5'-TCTGACCCTGCACTGAGTATTTC-3'  5'-ATCCATGACGCCTCTGTGC-3'  5'-GGTGCTTGGGATTTGTATGTTG -3'  5'-GTCTCAGTGCCCACATTATACAAG-3'  5'-AGTTGGGTTTGCTTCTCATCAC-3'  5'-CTGTCAACCAGCCGGATAAC-3'  5'-GACTTGTTGTTGGCGACTGC-3' |

**Supplementary Table 3**

Probe sequences of FISH assay

|  |  | | | **Probe sequences** |  |
| --- | --- | --- | --- | --- | --- |
| PVT1 | |  | 5’-TCCAGCTTTAGGTCACGTAAGGACA-3’  5’-GACTGGCTCTTAATTCTCCAATCTCA-3’  5’-CAGATGAACCAGGTGAACACAGAGC-3’  5’-GATCTATGGCATGGGCAGGGTAGA-3’  5’-CTAGCAGCAACAGGAGAAGCAAACA-3’  5’-GGGAGCCCGTTATTCTGTCCTTCT-3’  5’-GTGATCCTCTTGGTGGGGCTTGT-3’  5’-GTGAACTCCTCAGCCTCCAAGCGT-3’  5’-TTCATCCTGAGTCTCAAGATGCAGTA-3’  5’-CTCTTCAGGCCTCTTTGACAGCC-3’ | | |
